# Clinical Application Neutrophil Gelatinase-Associated Lipocalin and Kidney Injury Molecule-1 as Indicators of Inflammation Persistence and Acute Kidney Injury in Children with Urinary Tract Infection

**DOI:** 10.1155/2013/947157

**Published:** 2013-07-09

**Authors:** Stanislava Petrovic, Natasa Bogavac-Stanojevic, Amira Peco-Antic, Ivana Ivanisevic, Jelena Kotur-Stevuljevic, Dusan Paripovic, Miron Sopic, Zorana Jelic-Ivanovic

**Affiliations:** ^1^Department of Medical Biochemistry, Faculty of Pharmacy, University of Belgrade, P.O. Box 146, 11000 Belgrade, Serbia; ^2^School of Medicine, University of Belgrade, 11000 Belgrade, Serbia; ^3^Department of Nephrology, University Children's Hospital, 11000 Belgrade, Serbia

## Abstract

*Background*. The aim of this study was to examine the novel renal biomarkers neutrophil gelatinase-associated lipocalin (NGAL) and kidney injury molecule-1 (KIM-1) to assist pediatricians in the assessment of longer duration of inflammation and acute kidney injury (AKI) development during urinary tract infection (UTI). *Methods*. The patients enrolled in the study comprised 50 children (mean age was 6 months) with UTI. NGAL in serum and urine (sNGAL and uNGAL, resp.) and KIM-1 in urine were measured by enzyme-linked immunosorbent assays. *Results*. uNGAL levels in subjects with longer duration of inflammation were higher (115.37 ng/mL) than uNGAL levels in subjects with shorter duration of inflammation (67.87 ng/mL, *P* = 0.022). Difference in sNGAL and KIM-1 levels was not significant (*P* = 0.155 and *P* = 0.198, resp.). Significant difference was seen in KIM-1 excretion among groups with and without AKI (*P* = 0.038). KIM-1 was not able to discriminate between subjects with and without AKI (area under the curves (AUC) = 0.620, *P* = 0.175). *Conclusions*. uNGAL cannot be used for screening of the duration of inflammation during UTI. Accuracy of KIM-1 in screening of AKI development in children with UTI is low. We suggest larger studies to check the negative predictive value of KIM-1 for the development of AKI.

## 1. Introduction

Urinary tract infection (UTI) is one of the most common infectious diseases encountered by pediatric healthcare providers [1]. Early diagnosis and treatment of UTI are important, because missed or delayed diagnosis of UTI may result in the failure of appropriate treatment and possibly lead to long-term consequences, including renal scarring, hypertension, and chronic renal failure [2]. More severe forms of UTI may also cause acute complications. Children with UTI may develop a prerenal type of acute kidney injury (AKI) as a result of renal hypoperfusion due to severe dehydration. The cause of AKI in children with UTI may also be of a renal origin, but significantly lower. In any case, AKI should be discovered as soon as possible to start the therapy and prevent the progression and consequences (permanent damage to the kidneys).

The incidence of AKI in pediatrics is significant, and despite alarming data, therapeutic interventions have failed to effect a meaningful difference in outcomes [3]. At 3–5 years follow up, 40%–50% of pediatric patients who suffered AKI show signs of chronic renal insufficiency, indicating that sublethal injury permanently alters the renal bed [4]. Timely identification of children with AKI may be critical to management, though current laboratory and clinical markers may be unreliable for acute injury [3]. Several-fold changes in serum creatinine or creatinine clearance are the main criteria that have been used to diagnose AKI. However, changes in serum creatinine values lag behind the degree of injury to the renal tubular epithelial cells [3]. The search is on for real-time markers of AKI, which would allow for rapid and reliable diagnosis. A good AKI biomarker should be noninvasive, easily obtainable, and measurable using standardized assays with fast results and reasonable cost to perform [5]. Also, it is important to state that they change only because of kidney damage and not due to infection, inflammation, and other causes. Recently, several biomarkers have been explored for the early diagnosis of AKI, including cystatin C, interleukin-18 (IL-18), neutrophil gelatinase-associated lipocalin (NGAL), and kidney injury molecule-1 (KIM-1) [6].

Cystatin C is a cysteine protease inhibitor that is synthesized and released into the blood at a relatively constant rate by all nucleated cells, freely filtered by the glomerulus, completely reabsorbed by the proximal tubule, and not secreted [7]. In a number of baseline pediatric studies, serum cystatin C levels were diagnostically superior to serum creatinine and were independent of gender, body composition, or muscle mass [8]. However, clinicians should be cognizant of extrarenal conditions and pharmacological factors that can influence the results of serum cystatin C assays.

IL-18 is a proinflammatory cytokine that is induced and cleaved in the proximal tubule and subsequently easily detected in the urine following ischemic AKI [9]. Peak urine IL-18 concentration increases with worsening AKI severity in critically ill children but performs poorly as an early predictor of AKI [10]. Further studies will hopefully elucidate which patients and in what clinical settings an inflammatory marker such as IL-18 can be useful for early AKI prediction [5].

NGAL is a novel protein identified in human neutrophil granules and is considered to be a component of the innate immune system, highly involved in several cellular responses, including bacteriostasis, cell proliferation and differentiation, and apoptosis [11, 12]. NGAL is a 25 kDa lipocalin secreted by activated neutrophils, expressed in many cells, and is upregulated in several injury settings including infection, cancer and renal tubular injury [5]. Urine and plasma NGAL measurements represent early biomarkers of AKI in a heterogeneous pediatric intensive care setting, being able to predict this complication about 1-2 days prior to the rise in serum creatinine with high sensitivity [13, 14]. Therefore, NGAL has good features as a marker of renal function, but it is also a marker of inflammation so that the diagnosis of AKI is not applicable for all pathological conditions.

KIM-1 has been examined lately as a marker of kidney function, as well as its stability in different clinical conditions. KIM-1 is a type-1 transmembrane glycoprotein expressed in low levels of the normal kidney, which is highly upregulated in the proximal tubules after ischemic or toxic AKI [5]. With renal tubular injury, the extracellular domain of KIM-1 is cleaved from the transmembrane domain by proteolytic enzymes and released into the urine [15–17]. KIM-1 mediates epithelial phagocytosis in the injured kidney converting the proximal epithelial cell into a phagocyte, with potentially important pathophysiological implications for modulation of the immune response and repair process after injury [18]. 

However, none of these markers have been systematically evaluated in various clinical settings of AKI [19]. Furthermore, it is unlikely that a single biomarker will consistently provide sufficient precision to detect AKI early in all clinical situations; a combination of biomarkers could provide disease specific patterns or increased precision [20]. In our study, we examined the changes in parameters of renal function: KIM-1, urine, and serum level of NGAL (uNGAL and sNGAL, resp.) in relation to infection and in relation to the development of AKI. We also investigated the diagnostic ability to screen AKI development and longer duration of inflammation in children with UTI.

## 2. Patients and Methods

We performed a prospective single-center study of children with UTI at the University Children's Hospital in Belgrade, Serbia, over a period of one year. Fifty patients with first febrile UTI action at admission to the hospital were enrolled in this study. An informed consent of parents was obtained prior to enrolling children into the study, which was planned according to ethical guidelines following the Declaration of Helsinki. The institutional review committee approved our study protocol thereby following local biomedical research regulations. Children with known urogenital or anorectal malformations or neurological disease were excluded. Investigations, treatment, and further management were according to the hospital guidelines. After obtaining informed consent at the time of admission to the hospital, urine culture, urinalysis, serum urea, creatinine (Cr), C-reactive protein (CRP), and white blood cell (WBC) count measurements were performed. Patients who met the following criteria were included in the study: fever higher than 38.5°C with no other recognizable cause, leukocytosis defined as leukocyte count more than the normal value according to age, CRP higher than 20 mg/L, positive dipstick for leukocyte esterase, and/or pyuria (urine specimen with ≥10 WBC). Urine samples were obtained by midstream clean catch or sterile bags, and UTI was diagnosed if there was significant bacteriuria (>100.000 colony-forming units (CFU/mL)) in the urine culture. Urine samples were obtained in hospital by health care personnel, and the collection of data as well as laboratory methods were consistent through the study period. All children underwent abdominal and urinary tract ultrasound examination within 48 h of admission to the hospital while voiding cystourethrography (VCUG) was optional, at the treating physician's request and parents' decision. For all patients, repeat urine, urine culture, blood WBC, and CRP were performed after 48–72 h of admission to the hospital. Discharge criteria were evaluated by analyzing the clinical response to the antibiotics therapy (fever, blood WBC and CRP, urine WBC, and urine culture). 

The classification of patients was carried out in two ways: assessment of renal function and of the duration of inflammation at UTI according to the CRP value at discharge from hospital. Assessment of renal function was performed via estimated GFR using the Schwartz formula [21]. The division of patients into groups according to AKI development (with AKI and without AKI, resp.) was performed according to the decline of GFR to below 25% in relation with the normal values for age [22]. In another classification, patients were divided according to CRP value at discharge from the hospital. CRP is a direct and quantitative marker of the acute phase reaction, and the severity of inflammation at UTI, as measured by serum CRP, is significantly associated with permanent renal damage. A level of CRP ≥10 mg/L is considered to be indicative of a clinically-relevant inflammatory condition [23], while high levels of CRP mean prolonged inflammation that can lead to the development of fibrosis and scar formation in the kidney. Accordingly, two groups were formed: patients with CRP levels higher than 10 mg/L were marked as patients with a longer duration of inflammation (coded with 1), while patients with CRP levels less than 10 mg/L were marked as patients with a shorter duration of inflammation (coded with 0). 

Blood and urine samples were drawn from each patient to measure the following parameters: serum concentrations of NGAL, Cr, urea and CRP, urine concentrations of NGAL, and KIM-1. The urine samples were immediately centrifuged at 4°C for 15 min at 13,000 g. Samples were frozen at −80°C and analysed within 3 months. Cr and urea levels were analyzed by routine methods (Olympus System Reagents using an Olympus analyser AU 2700, Hamburg, Germany). CRP was determined by a nephelometric method (Siemens C-reactive protein assay using a Dimension RxL Max Integrated Chemistry System, Erlangen, Germany). sNGAL, uNGAL, and KIM-1 were measured by enzyme-linked immunosorbent assays (ELISAs) using polyclonal goat antibodies against human proteins coated onto the wells of microtitre plates (R&D Systems Europe, Abingdon, United Kingdom). Urine samples were diluted 1 : 60 for uNGAL, while serum samples for sNGAL were diluted 1 : 40. Urine samples for KIM-1 were not diluted. NGAL and KIM-1 levels were expressed as nanograms per milliliter.

Statistical analyses were made with MedCalc for Windows Version 9.6.3. (Mariakerke, Belgium) statistical software. The distributions of variables were checked for normality, and logarithmic transformation was used for skewed data. Interindividual and intraindividual variation was calculated in groups of subjects formed according to ages. The coefficient of variation (CV) between subjects (interindividual variation) for each parameter was calculated at the time of admission to the hospital and at the time of hospital discharge. Intraindividual variation was expressed as the correlation between the value measured by each subject at the time of admission to the hospital and value of the same parameter at the time of hospital discharge, by calculating Spearman's correlation coefficient. Differences between subjects at the time of admission to the hospital and at the time of hospital discharge (two points of measurement) were analyzed with a paired Student *t*-test. The effects of inflammation and AKI on serum and urinary variables at the time of hospital discharge were compared by analysis of covariance (ANCOVA) with adjustment for baseline levels (values at the time of admission to the hospital). Additionally, change in examined variables was analysed using repeated-measures analysis of variance (ANOVA) with “time” as within and the “presence of AKI” as between factors. Accuracy of the examined parameters was assessed using receiving operative characteristic (ROC) curve analysis. Parameters with significant accuracy were combined with other parameters, and curves for these models were plotted, and areas under the curves (AUCs) were presented as C statistics from the analysis. Data are shown as mean ± standard deviation for normally-distributed continuous variables and as relative of absolute frequencies for categorical variables. Logarithmic-transformed variables were expressed as geometrical mean and 95% confidence interval (CI) for the mean. All tests were considered significant at *P* < 0.05. 

## 3. Results

The patients enrolled in the study comprised 50 Caucasian children from 1 month to 12 years of age with first-time community-acquired UTI, of whom 28 were male and 22 were females. Eighty-six percent of children were younger than 12 months, 8% were from 12 months to 36 months of age, and 6% were older than 36 months. As might be presumed, in most cases the cause of UTI was *Escherichia coli* (88%, 44 cases). The average hospital length of stay was 5.5 days. Baseline characteristics of the study population are presented in Table 1. 

**Figure 1:**
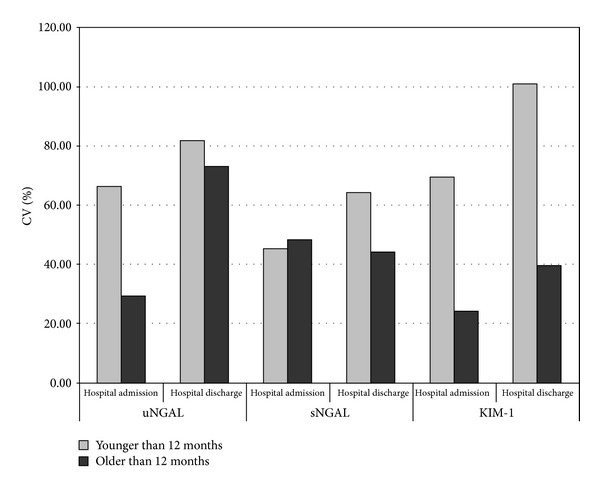
Interindividual variability: comparison of coefficient of variation for uNGAL, sNGAL and KIM-1 in younger and older subjects at the time of admission to the hospital and at the time of hospital discharge.

Because the age of the children was from 1 month to 12 years, inter- and intraindividual variability in each measured parameter was calculated at the time of admission to the hospital and at the time of hospital discharge, separately in the groups of children younger and older than 12 months. Interindividual variability was presented in Figure 1. In groups of older children, CVs for all parameters (except for sNGAL at the time of admission to the hospital) were lower than CVs in groups of younger children. Likewise, CVs were higher in all groups at the time of hospital discharge than at the time of admission to the hospital (except for sNGAL in group of older children). Correlation coefficients (intraindividual variability) for each parameter in younger and older subjects when measured at the time of admission to the hospital and at the time of hospital discharge were significant for uNGAL in younger subjects (*r* = 0.736, *P* < 0.001) and for sNGAL in older subjects (*r* = 0.786, *P* = 0.036). The agreement between values measured at the time of admission to the hospital and at the time of hospital discharge for other parameters was lower and without statistical significance. There was no difference in examined parameters (uNGAL, sNGAL, and KIM-1) between children younger than 12 months and children older than 12 months (data not shown).

Renal biomarkers and inflammatory parameters of subjects at the time of admission to the hospital and at the time of hospital discharge are listed in Table 2. The levels of all parameters were significantly higher at the time of admission to the hospital than corresponding values at the time of hospital discharge. 

Effects of infection (expressed in relation to the concentration of CRP) on levels of renal parameters are presented in Table 3. Adjusting by ANCOVA for values at the time of admission to the hospital, uNGAL levels in subjects with longer duration of inflammation were higher (115.37 ng/mL) than uNGAL levels in subjects with shorter duration of inflammation at the same time (67.87 ng/mL, *P* = 0.022). After inclusion of ages as additional covariate difference in uNGAL values between groups was significant, *P* = 0.031. sNGAL and urinary KIM-1 levels showed not significant (*P* = 0.155 and *P* = 0.198, resp.) trends towards increases in the group with longer duration of inflammation compared with the group with shorter duration of inflammation. 

**Figure 2:**
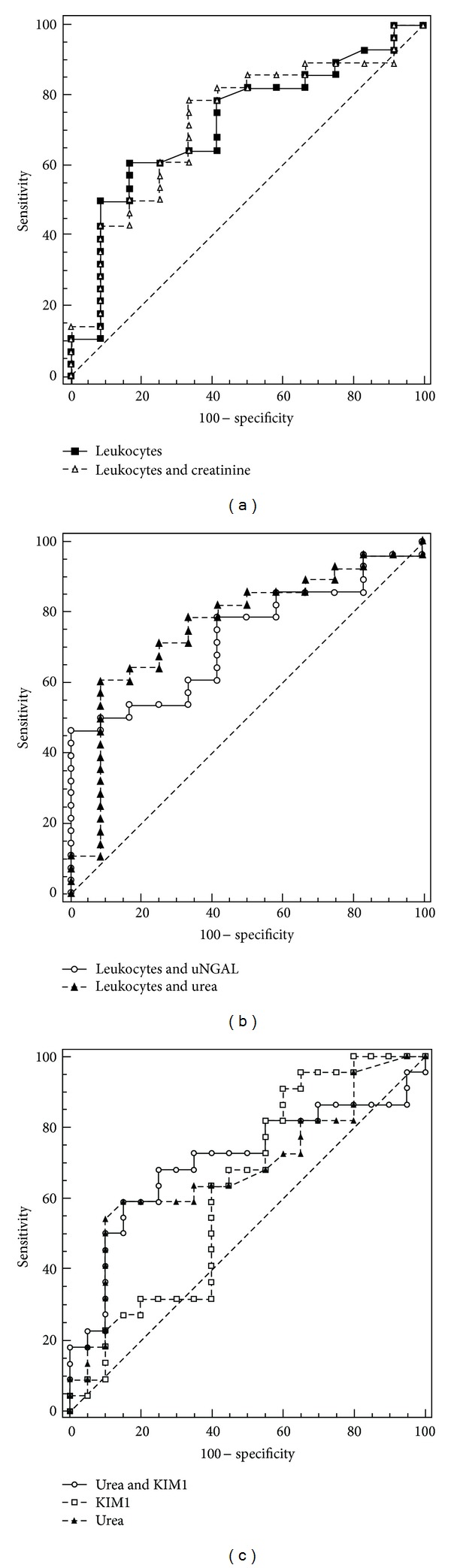
ROC curves for parameters with significant diagnostic ability for duration of inflammation and significant diagnostic ability for AKI. (a): ROC curves for a number of leukocytes and for a combination of leukocytes with Cr for detection of longer duration of inflammation; (b): ROC curves for a combination of leukocytes with urea or uNGAL for detection of longer duration of inflammation; (c): ROC curves for parameters with significant discriminative abilities for AKI (KIM-1, urea, combination of KIM-1 and urea).

The ability of renal parameters to detect longer duration of inflammation was investigated by ROC curve analysis. Urinary KIM-1 (AUC = 0.519, *P* = 0.847), uNGAL (AUC = 0.549, *P* = 0.638), sNGAL (AUC = 0.516, *P* = 0.855), Cr (AUC = 0.538, *P* = 0.695), and urea (AUC = 0.560, *P* = 0.538) at the time of admission to the hospital were not able to detect longer duration of inflammation. Of the parameters we examined, only a number of leukocytes significantly detected longer duration of inflammation (AUC = 0.688, *P* = 0.047), (Figure 2(a)). We also investigated the potential benefit of adding renal parameters to a number of leukocytes to better discriminate subjects with longer duration of inflammation from subjects with shorter duration of inflammation. The addition of Cr and urea increased the AUC for number of leukocytes (AUC = 0.723, *P* = 0.027 (Figure 2(a)) and AUC = 0.762, *P* = 0.009 (Figure 2(b)), resp.), whereas the addition of uNGAL had a marginal effect (AUC = 0.695, *P* = 0.041), (Figure 2(b)).

**Figure 3:**
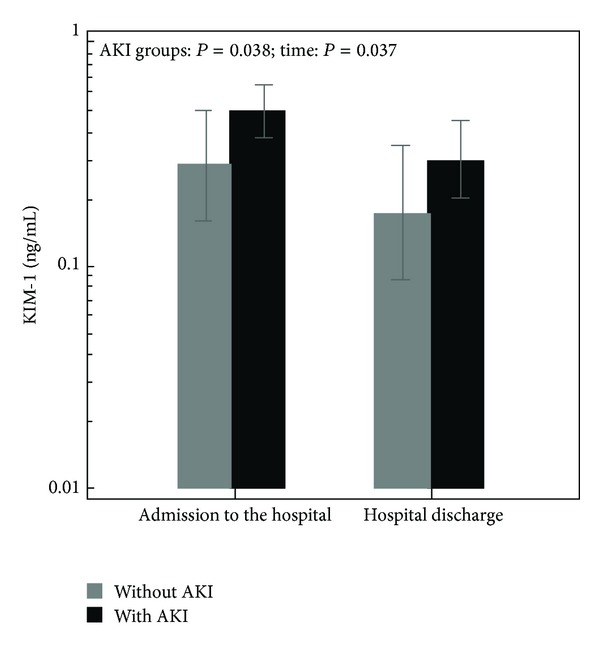
Urinary KIM-1 levels at the time of subject admission to the hospital and at the time of hospital discharge among groups with AKI and without AKI. In the figure, there are presented means and standard error values.

Time-related changes in urinary KIM-1 excretion differed significantly among two points of measurement (at the time of hospital admission and at the time of hospital discharge), and a similar significant difference was seen in urinary KIM-1 excretion among groups with AKI and without AKI (AKI groups: *P* = 0.038; time: *P* = 0.037; AKI group × time: *P* = 0.971; repeated-measures ANOVA, Figure 3). 

The ability of KIM-1 to detect AKI development was investigated by ROC curve analysis. Urinary KIM-1 at the time of subject admission to hospital (AUC = 0.620, *P* = 0.175) was not able to discriminate between subjects with AKI and without AKI. On the other hand, the AUC for urea was higher (AUC = 0.681, *P* = 0.040). A combination of KIM-1 with urea increased the ability of urea to discriminate impaired risk for AKI (AUC = 0.705, *P* = 0.023), (Figure 2(c)).

## 4. Discussion

All recent pediatric AKI studies demonstrate that AKI results more often as a sequel to another systemic illness or its treatment [24, 25], and not from primary kidney disease itself [20]. A possible cause for the development of AKI is a severe form of UTI. Late AKI diagnosis and delay in supportive therapy may result in increased mortality. This is supported by the observation that even small increases in Cr (50% or 0.3 mg/dL) are independently associated with patient mortality [26–28]. It is clear that while the RIFLE (risk, injury, failure, loss, end-stage kidney disease) [27] and AKIN (acute kidney injury network) [29] strata are helpful for retrospective reviews and epidemiological study, they have limited utility to the clinician evaluating a child in real-time [3]. On the other hand, current diagnostic procedure for monitoring renal involvement during UTI (upper UTI) and related parenchymal inflammation is scintigraphy. This is an invasive and expensive technique that is not always available, and a search for noninvasive methods is therefore relevant [30]. Substantial research has been carried out over the past decade to discover and validate biomarkers to detect AKI before a rise in Cr in hospitalized patients [20]. 

Taking into account the data above, the aim of this study was to examine the novel renal biomarkers NGAL and KIM-1 to assist pediatricians in the assessment of longer duration of inflammation and developing AKI during UTI. However, the first step was to examine the stability of examined parameters in infection. The levels of uNGAL were significantly higher at the time of admission to the hospital than corresponding values at the time of hospital discharge, as well in subjects with longer duration of inflammation than in subjects with shorter duration of inflammation; so our results confirmed that uNGAL is sensitive to the state of infection.

According to the literature, both uNGAL and uNGAL/Cr can be used as sensitive markers for early prediction of UTI in the absence of AKI and chronic kidney disease [1]. NGAL is released by activated neutrophils of the infected host and prevents bacterial iron uptake consuming the ambient iron and thus reducing bacterial growth [7, 31]. Expression of NGAL increases as part of the immune response to remove bacteria in the early stage of infection [1]. Also, uNGAL is increased in AKI induced by nephrotoxins or ischemia, probably because NGAL expression is induced to contribute to tissue regeneration after kidney damage [32, 33]. However, in this case, NGAL measurements may be influenced by a number of coexisting variables, such as pre-existing renal disease [34] and systematic or urinary tract infections [35, 36].

In addition, we tested whether uNGAL could be a reliable marker for discrimination between longer and shorter duration of inflammation in the assessment of disease severity. Of the parameters KIM-1, uNGAL, sNGAL, Cr, and urea, only the number of leukocytes significantly detected a longer duration of inflammation (AUC = 0.688, *P* = 0.047). We also investigated the potential benefit of adding renal parameters to the number of leukocytes to better discriminate subjects with longer duration of inflammation. The addition of Cr and urea increased the AUC for a number of leukocytes (AUC = 0.723, *P* = 0.027 and AUC = 0.762, *P* = 0.009, resp.), whereas the addition of uNGAL had a marginal effect (AUC = 0.695, *P* = 0.041), (Figures 2(a) and 2(b)). The baseline value of uNGAL was not able to discriminate between children who will have a longer duration of inflammation than children with a shorter duration of inflammation. 

Although the levels of KIM-1 were higher at the time of admission to the hospital than corresponding values at the time of hospital discharge, KIM-1 showed no significant trends towards increases in the group with longer duration of inflammation compared with the group with shorter duration of inflammation. Herein is reviewed the diagnostic and prognostic performance of several types of urinary biomarkers for the diagnosis and risk stratification of AKI, where KIM-1 was not classified in the group of inflammatory markers for the diagnosis of AKI, in contrast to NGAL and IL-18 [37]. For now, it is well known that KIM-1 expression is elevated in various etiologies of AKI, chronic kidney disease, the kidney transplant population, and renal cell carcinoma [38]. Previous studies have shown that the normalized urinary KIM-1 levels were significantly higher in patients with ischemic acute tubular necrosis compared to levels in patients with other forms of acute renal failure or chronic renal disease [16], and in patients with AKI than in patients with UTI [19]. Existing studies have been insufficiently powered to establish a cut-off value that is predictive of AKI [38]. Furthermore, it is unclear whether expression of KIM-1 is related to the pathogenesis of the injury itself or marker of attempted recovery and repair. KIM-1 is unique in being the first molecule that transforms kidney proximal epithelial cells into semiprofessional phagocytes [39]. As a consequence of its role in enhancing the clearance of dead cells by the surviving tubular cells, KIM-1 may act to modulate immune response in AKI, and phagocytosis of apoptotic cells may downregulate the proinflammatory immune response [18].

 We tested the hypothesis that urinary excretion of KIM-1 is increased in children with UTI, related to renal damage. We wanted to investigate the ability of this protein as a potential biomarker for the diagnosis of AKI in the presence of UTI. Our study showed significant difference in urinary KIM-1 excretion among groups with AKI and without AKI (Figure 3), but urinary KIM-1 at the time of hospital admission was not able to discriminate between these two groups of patients (AUC = 0.620, *P* = 0.175). The discrimination ability of KIM-1 for AKI was less than the traditional parameter urea (AUC = 0.681, *P* = 0.040). A combination of KIM-1 with urea increased the ability of urea to discriminate impaired risk for AKI (AUC = 0.705, *P* = 0.023), (Figure 2(c)). 

The published clinical studies of urine KIM-1 are small so far, and there is no clear evidence whether urinary KIM-1 is an effective AKI diagnostic test in humans [38]. Early studies of adults suggest that KIM-1 appears to discriminate patients with different types of acute tubular necrosis (hospitalized patients, critically ill patients, patients with acute graft rejection) from those without AKI [19, 40]. To our knowledge, this is the first study where KIM-1 is considered as a biomarker for prediction of AKI in children with UTI. The first reported pediatric study in 40 children undergoing cardiac surgery revealed that urine KIM-1 at 12 h postoperative had an AUC of 0.83 for detection of subsequent AKI [19]. A similar result for the accuracy of KIM-1 in the prediction of AKI to our study was obtained in a study which included 252 children (mean age 11.4 ± 4.8 years) with various primary organ systemic diagnoses. In this study, AUC for KIM-1 to predict AKI was 0.61 (95% CI 0.48–0.73) [20]. Patients were classified by the Cr-based pediatric modified RIFLE (pRIFLE), and KIM-1 had a higher AUC for predicting the presence of pRIFLE-I AKI than for predicting pRIFLE-R AKI. Based on this results and results from our study, KIM-1 could serve as AKI biomarker in more severely-ill children. 

The limitations of our study are the small study population, and therefore small numbers of patients in groups with AKI and without AKI, and the absence of data controls. However, because of the design of the study, formation of a control group was not necessary. Furthermore, the lack of data from a dimercaptosuccinic acid (DMSA) scan is not critical, because the main aim of this study was to investigate the stability of KIM-1 as a AKI biomarker in a state of inflammation in children with UTI. 

## 5. Conclusions 

Our findings suggest that although uNGAL is suitable for early diagnosis of UTI, it cannot be used for screening of the duration of inflammation during UTI or the severity of the illness. On the other hand, KIM-1 is less sensitive to the state of inflammation than NGAL and hence more specific to ischemic kidney injury, accuracy of KIM-1 in screening of AKI development in children with UTI is not so good. But for now, it should not be dismissed that KIM-1 is a potential biomarker for AKI in the pediatric population with UTI, since it is used from a urine sample instead of blood, and the ongoing development of rapid screening urine test strips. However, we should consider the implementation of a comprehensive study that would examine the negative predictive value of KIM-1 in AKI development during UTI and developing an algorithm that would greatly facilitate the work of pediatricians. 

## Figures and Tables

**Table 1:** Demographic and biochemical characteristics in the study population.

	All subjects (*n* = 50)	Without AKI (*n* = 28)	With AKI (*n* = 22)
Age (months)*	6.00 (4.00–7.00)	3.3 (2.37–4.40)	11.7 (7.07–18.12)
Gender, male (%)	56	61.5	38.5
BMI (kg/m^2^)	11.67 ± 4.29	10.36 ± 2.02	12.88 ± 5.69^a^
Fever (°C)	39.22 ± 0.658	39.17 ± 0.58	39.22 ± 0.77
Duration of fever (days)	3.1 ± 1.45	2.74 ± 1.29	3.75 ± 1.52^a^
Duration in hospital (days)*	5.46 (5.00–6.00)	5.41 (4.24–6.90)	5.89 (5.21–7.61)
GFR (mL/min/m^2^)	49.20 ± 13.34	51.13 ± 10.07	40.42 ± 12.49
sCr (*µ*mol/L)	57.85 ± 13.99	50.70 ± 7.56	65.40 ± 15.60
Urea (mmol/L)*	2.87 (2.50–3.31)	2.47 (2.06–2.98)	3.40 (2.61–4.42)^a^
Leukocyte number (×10e9/L)	17.55 ± 5.92	16.38 ± 5.16	19.12 ± 6.5^a^
Urine culture (%)			
Escherichia coli	88	90.9	85.0
Klebsiella	4.8	4.5	5
Proteus	4.8	0	10
Other	2.4	4.5	0
Therapy (%)			
Longaceph (ceftriaxone)	38.6	39.1	38.1
Amikacin	36.4	30.4	42.9
Combination	25.0	30.5	19.0

Data are mean ± SD, percentages, or *geometrical mean (95% CI for the mean) for variables with skewed distribution. ^a^significantly different from group without AKI by Student *t*-test.

**Table 2:** Renal biomarkers and inflammatory parameters of 50 subjects at the time of admission to the hospital and at the time of hospital discharge.

	Hospital admission	Hospital discharge	*P *
sNGAL, ng/mL	153.90 ± 75.04	91.34 ± 56.63	<0.001
uNGAL, ng/mL	155.00 ± 92.39	94.03 ± 76.76	<0.001
KIM-1, ng/mL	0.38 (0.28–0.50)	0.24 (0.17–0.34)	0.037
CRP, mg/L	72.62 (54.38–96.96)	13.12 (9.71–17.72)	<0.001

Data are mean ± SD or geometrical mean (95% CI for the mean) for variables with skewed distribution.

Serum level of neutrophil gelatinase-associated lipocalin (sNGAL), urine level of neutrophil gelatinase-associated lipocalin (uNGAL), urine level of kidney injury molecule-1 (KIM-1), and serum level of C-reactive protein (CRP).

**Table 3:** Effects of infection on renal parameter levels at the time of hospital discharge when values at the time of admission to the hospital were used as covariates.

	Shorter duration of inflammation *n* = 17	Longer duration of inflammation *n* = 33	*P *
sNGAL, ng/mL	78.80 ± 14.68	104.82 ± 10.17	0.155
uNGAL, ng/mL	67.87 ± 16.04	115.37 ± 11.72	0.022^a^
KIM-1, ng/mL	0.169 (0.08–0.34)	0.295 (0.182–0.479)	0.198

Data are adjusted mean ± SD or geometrical mean (95% CI for the mean) for variables with skewed distribution. ^a^significant differences between groups in ANCOVA with adjustment for baseline values.

Shorter duration of inflammation—CRP level less than 10 mg/L at the time of hospital discharge, longer duration of inflammation—CRP level higher than 10 mg/L at the time of hospital discharge.

## References

[1] Yilmaz A, Sevketoglu E, Gedikbasi A (2009). Early prediction of urinary tract infection with urinary neutrophil gelatinase associated lipocalin. *Pediatric Nephrology*.

[2] Hansson S, Jodal U, Avner ED, Harmon WEB, Niaudet P (2004). Urinary tract infection. *Pediatric Nephrology*.

[3] Basu RK, Wheeler D (2011). Approaches to the management of acute kidney injury in children. *Recent Patents on Biomarkers*.

[4] Askenazi DJ, Feig DI, Graham NM, Hui-Stickle S, Goldstein SL (2006). 3–5 year longitudinal follow-up of pediatric patients after acute renal failure. *Kidney International*.

[5] Al-Ismaili Z, Palijan A, Zappitelli M (2011). Biomarkers of acute kidney injury in children: discovery, evaluation, and clinical application. *Pediatric Nephrology*.

[6] Han WK, Bonventre JV (2004). Biologic markers for the early detection of acute kidney injury. *Current Opinion in Critical Care*.

[7] Nguyen MT, Devarajan P (2008). Biomarkers for the early detection of acute kidney injury. *Pediatric Nephrology*.

[8] Zaffanello M, Franchini M, Fanos V (2007). Review: is serum cystatin-C a suitable marker of renal function in children?. *Annals of Clinical and Laboratory Science*.

[9] Melnikov VY, Ecder T, Fantuzzi G (2001). Impaired IL-18 processing protects caspase-1-deficient mice from ischemic acute renal failure. *Journal of Clinical Investigation*.

[10] Washburn KK, Zappitelli M, Arikan AA (2008). Urinary interleukin-18 is an acute kidney injury biomarker in critically ill children. *Nephrology Dialysis Transplantation*.

[11] Devarajan P (2008). Neutrophil gelatinase-associated lipocalin—an emerging troponin for kidney injury. *Nephrology Dialysis Transplantation*.

[12] Kjeldsen L, Bainton DF, Sengelov H, Borregaard N (1993). Structural and functional heterogeneity among peroxidase-negative granules in human neutrophils: identification of a distinct gelatinase-containing granule subset by combined immunocytochemistry and subcellular fractionation. *Blood*.

[13] Zappitelli M, Washburn KK, Arikan AA (2007). Urine neutrophil gelatinase-associated lipocalin is an early marker of acute kidney injury in critically ill children: a prospective cohort study. *Critical Care*.

[14] Wheeler DS, Devarajan P, Ma Q (2008). Serum neutrophil gelatinase-associated lipocalin (NGAL) as a marker of acute kidney injury in critically ill children with septic shock. *Critical Care Medicine*.

[15] Ichimura T, Bonventre JV, Bailly V (1998). Kidney injury molecule-1 (KIM-1), a putative epithelial cell adhesion molecule containing a novel immunoglobulin domain, is up-regulated in renal cells after injury. *Journal of Biological Chemistry*.

[16] Han WK, Bailly V, Abichandani R, Thadhani R, Bonventre JV (2002). Kidney Injury Molecule-1 (KIM-1): a novel biomarker for human renal proximal tubule injury. *Kidney International*.

[17] Bailly V, Zhang Z, Meier W, Cate R, Sanicola M, Bonventre JV (2002). Shedding of kidney injury molecule-1, a putative adhesion protein involved in renal regeneration. *Journal of Biological Chemistry*.

[18] Bonventre JV, Yang L (2010). Kidney injury molecule-1. *Current Opinion in Critical Care*.

[19] Han WK, Waikar SS, Johnson A (2008). Urinary biomarkers in the early diagnosis of acute kidney injury. *Kidney International*.

[20] Du Y, Zappitelli M, Mian A (2011). Urinary biomarkers to detect acute kidney injury in the pediatric emergency center. *Pediatric Nephrology*.

[21] Schwartz GJ, Muñoz A, Schneider MF (2009). New equations to estimate GFR in children with CKD. *Journal of the American Society of Nephrology*.

[22] Langlois V, Geary DF, Schaefer F (2006). Laboratory evaluation at different ages. *Comprehensive Pediatric Nephrology*.

[23] Pearson TA, Mensah GA, Alexander RW (2003). Markers of inflammation and cardiovascular disease: application to clinical and public health practice: a statement for healthcare professionals from the centers for disease control and prevention and the American Heart Association. *Circulation*.

[24] Williams DM, Sreedhar SS, Mickell JJ, Chan JCM (2002). Acute kidney failure: a pediatric experience over 20 years. *Archives of Pediatrics and Adolescent Medicine*.

[25] Hui-Stickle S, Brewer ED, Goldstein SL (2005). Pediatric ARF epidemiology at a tertiary care center from 1999 to 2001. *American Journal of Kidney Diseases*.

[26] Price JF, Mott AR, Dickerson HA (2008). Worsening renal function in children hospitalized with decompensated heart failure: evidence for a pediatric cardiorenal syndrome?. *Pediatric Critical Care Medicine*.

[27] Akcan-Arikan A, Zappitelli M, Loftis LL, Washburn KK, Jefferson LS, Goldstein SL (2007). Modified RIFLE criteria in critically ill children with acute kidney injury. *Kidney International*.

[28] Plötz FB, Bouma AB, Van Wijk JAE, Kneyber MCJ, Bökenkamp A (2008). Pediatric acute kidney injury in the ICU: an independent evaluation of pRIFLE criteria. *Intensive Care Medicine*.

[29] Mehta RL, Kellum JA, Shah SV (2007). Acute kidney injury network: report of an initiative to improve outcomes in acute kidney injury. *Critical Care (London, England)*.

[30] Andersson L, Preda I, Hahn-Zoric M (2009). Urinary proteins in children with urinary tract infection. *Pediatric Nephrology*.

[31] Berger T, Togawa A, Duncan GS (2006). Lipocalin 2-deficient mice exhibit increased sensitivity to Escherichia coli infection but not to ischemia-reperfusion injury. *Proceedings of the National Academy of Sciences of the United States of America*.

[32] Ichino M, Kuroyanagi Y, Kusaka M (2009). Increased urinary neutrophil gelatinase associated lipocalin levels in a rat model of upper urinary tract infection. *Journal of Urology*.

[33] Hirsch R, Dent C, Pfriem H (2007). NGAL is an early predictive biomarker of contrast-induced nephropathy in children. *Pediatric Nephrology*.

[34] Mitsnefes MM, Kathman TS, Mishra J (2007). Serum neutrophil gelatinase-associated lipocalin as a marker of renal function in children with chronic kidney disease. *Pediatric Nephrology*.

[35] Pisitkun T, Johnstone R, Knepper MA (2006). Discovery of urinary biomarkers. *Molecular and Cellular Proteomics*.

[36] Xu S, Venge P (2000). Lipocalins as biochemical markers of disease. *Biochimica et Biophysica Acta*.

[37] Coca SG, Parikh CR (2008). Urinary biomarkers for acute kidney injury: perspectives on translation. *Clinical Journal of the American Society of Nephrology*.

[38] Fontanilla J, Han WK (2011). Kidney injury molecule-1 as an early detection tool for acute kidney injury and other kidney diseases. *Expert Opinion on Medical Diagnostics*.

[39] Ichimura T, Asseldonk EJPV, Humphreys BD, Gunaratnam L, Duffield JS, Bonventre JV (2008). Kidney injury molecule-1 is a phosphatidylserine receptor that confers a phagocytic phenotype on epithelial cells. *Journal of Clinical Investigation*.

[40] van Timmeren MM, van den Heuvel MC, Bailly V, Bakker SJL, van Goor H, Stegeman CA (2007). Tubular kidney injury molecule-1 (KIM-1) in human renal disease. *Journal of Pathology*.

